# Ultrasound-Triggered Liposomes Encapsulating Quantum Dots as Safe Fluorescent Markers for Colorectal Cancer

**DOI:** 10.3390/pharmaceutics13122073

**Published:** 2021-12-03

**Authors:** Nahid S. Awad, Mohamed Haider, Vinod Paul, Nour M. AlSawaftah, Jayalakshmi Jagal, Renu Pasricha, Ghaleb A. Husseini

**Affiliations:** 1 Department of Chemical Engineering, Collage of Engineering, American University of Sharjah, Sharjah P.O. Box 26666, United Arab Emirates; nawad@aus.edu (N.S.A.); b00068146@aus.edu (V.P.); g00051790@alumni.aus.edu (N.M.A.); 2 Department of Pharmaceutics and Pharmaceutical Technology, College of Pharmacy, University of Sharjah, Sharjah P.O. Box 27272, United Arab Emirates; mhaider@sharjah.ac.ae; 3 Department of Pharmaceutics and Industrial Pharmacy, Faculty of Pharmacy, Cairo University, Cairo P.O. Box 71526, Egypt; 4 Materials Science and Engineering Program, American University of Sharjah, Sharjah P.O. Box 26666, United Arab Emirates; 5 Research Institute for Medical and Health Sciences, University of Sharjah, Sharjah P.O. Box 27272, United Arab Emirates; jayajagal@gmail.com; 6 Core Technology Platforms Operations, New York University Abu Dhabi, Abu Dhabi P.O. Box 129188, United Arab Emirates; renu.pasricha@nyu.edu

**Keywords:** quantum dots, liposomes, low-frequency ultrasound, controlled release

## Abstract

Quantum dots (QDs) are a promising tool to detect and monitor tumors. However, their small size allows them to accumulate in large quantities inside the healthy cells (in addition to the tumor cells), which increases their toxicity. In this study, we synthesized stealth liposomes encapsulating hydrophilic graphene quantum dots and triggered their release with ultrasound with the goal of developing a safer and well-controlled modality to deliver fluorescent markers to tumors. Our results confirmed the successful encapsulation of the QDs inside the core of the liposomes and showed no effect on the size or stability of the prepared liposomes. Our results also showed that low-frequency ultrasound is an effective method to release QDs encapsulated inside the liposomes in a spatially and temporally controlled manner to ensure the effective delivery of QDs to tumors while reducing their systemic toxicity.

## 1. Introduction

The application of nanomaterials in the drug delivery field has shown a rapid increase in the last decade, owing to their efficiency in cancer treatment and diagnosis. Developing a single multifunctional nanoscale drug delivery system with imaging, diagnostic and targeting capabilities is an active research field. Quantum dots are semiconductor nanoparticles with diameters in the range of 1 to 10 nm. Their small size results in unique optical characteristics, giving them promising potentials as novel fluorescent probes for diagnostic purposes [1,2]. QDs have a long fluorescent life with high stability against photobleaching. They also have broad absorption and narrow emission spectra, which allow detecting multiple colors of QDs following illumination with a single light source [3]. The special properties of QDs present them as attractive contenders for fluorescent imaging in the medical field, with many in vitro and in vivo studies investigating their use as optical probes for tumor imaging and monitoring of cancer development [4,5,6]. However, the use of QDs for intracellular labeling is associated with several challenges. Their low biocompatibility, diminished stability and high toxicity due to aggregation and surface degradation are critical issues limiting their medical applications and their use in humans [7,8]. Diverse methods have been investigated to overcome these limitations. One of these methods is the functionalization of QDs with different types of molecules such as antibodies, peptides and polymers [4,9]. However, since the photophysical properties of the QDs are directly related to surface states, surface modification using these ligands often results in quenching their fluorescence and decreasing their photostability [10,11]. Therefore, it is essential to reduce the interference with their surface while maintaining their electronic passivation. Addressing these issues has now become a priority to assess the clinical and bionanotechnology prospective of QDs.

A plausible solution to overcome the shortfall of QDs and enhance their intracellular delivery is to encapsulate them inside nanocarriers such as liposomes. Liposomes are nanosized (50 nm to 700 nm) artificial vesicles of spherical shape surrounded by a natural, nontoxic phospholipid bilayer and cholesterol resembling cellular membranes [12]. This structure allows the liposomes to be loaded with hydrophilic drugs (inside their cores) and hydrophobic drugs (inside their lipid bilayer). A polymer, poly (ethylene glycol) (PEG), can be added to their surface to form stealth liposomes. This will significantly increase their circulation time in the blood and reduce their elimination by the immune system [13]. The defective and leaky architecture of the tumor vasculature allows these liposomes to pass through and accumulate inside the tumor due to their small size. This is known as the enhanced permeability and retention (EPR) effect and plays an important role in delivering nanocarriers to tumors [14]. Therefore, nanocarriers, such as liposomes, are capable of selectively delivering high dosages of drugs to the tumor tissues without harming healthy, drug-sensitive tissues such as the heart tissue [15].

Quantum dots-liposomes combination (QD-liposomes) will enhance the biocompatibility and reduce the toxicity of QDs, paving the way for developing a novel hybrid nanoscale delivery system where both therapeutic and imaging agents can be codelivered. This can be achieved by encapsulating both therapeutic agents and QDs inside the liposomes, allowing a safer delivery to tumors while providing a clear in vivo monitoring of drug biodistribution to determine the progress and efficiency of the different neoplastic drugs during treatment. Several studies have shown a successful encapsulation of QDs either inside the bilayer membrane or in the core of the liposomes encapsulating therapeutic agents. These studies showed safer drug delivery and optical tracking, thus providing opportunities to create an effective theranostic nanoscale delivery system, where both diagnostic and therapeutic functions can be given in a single dose [16,17,18,19].

Following the accumulation of the QD-liposomes at the tumor site, it is crucial to ensure an effective and controlled release of the loaded QDs and their uptake by the cancer cells. Cell membranes represent a physical barrier to transport molecules into cells. One of the best methods to enhance cellular transport across cellular membranes is through the use of ultrasound. This is because ultrasonic waves propagate in soft tissues. When applied at the tumor site, stable cavitation or inertial cavitation can generate a local steady or transient ‘microstreaming’ flow that produces holes by shearing or stretching the cell membrane. This results in forming transient or permanent pores in the walls of the cancer cell membranes and their surrounding vasculature, thus enhancing the permeability of these walls, a process known as “sonoporation.” This will lead to enhancing the uptake of different types of molecules into the cancer cells. Although thermal and chemical effects might be produced as well, ultrasound enhancement of cellular permeability is mainly achieved through the mechanical effect (sonoporation), which makes it applicable to different molecules and cell types [20]. Having similar structures to cellular membranes, liposomal membranes are also subject to the sonoporation effect under the action of ultrasound. Thus, releasing their loads in a spatially and temporally controlled manner. This ensures a confined delivery of their loads to targeted tumors with a high degree of precision while minimizing adverse effects.

Our previous in vitro studies have shown that applying low-frequency ultrasound (LFUS) enhances both drug release from the liposomes and their uptake by the cancer cells [21,22,23,24,25]. In this study, we investigate the use of liposomes as a carrier delivering green graphene quantum dots to cancer cells and the effect of applying LFUS in enhancing the cellular uptake of free QDs and QDs loaded inside the liposomes by enhancing the permeability of both cellular and liposomal membranes.

## 2. Materials and Methods

### 2.1. Materials

Dipalmitoylphosphatidyl choline (DPPC) and 1,2-distearoyl-sn-glycero-3-phosphoethanolamine-N [amino (polyethylene glycol)-2000] (DSPE-PEG (2000)-NH_2_) were obtained from Avanti Polar Lipids Inc. (Alabaster, AL, USA, supplied by Labco LLC. Dubai, UAE). Cholesterol was obtained from Sigma Aldrich Chemie GmbH (Munich, Germany, supplied by Labco LLC. Dubai, UAE). Chloroform was obtained from Panreac Quimica S.A. (Barcelona, Spain). Graphene quantum dots solution was obtained from ACS Material (Pasadena, CA). The Avanti Mini Extruder extrusion kit is purchased from Avanti Polar Lipids Inc. (Alabaster, AL, USA). The 0.2 μm polycarbonate membranes and filter supports were obtained from Whatman PLC (Maidstone, England, UK). The human colorectal carcinoma (HCT116) cell line was purchased from the European Collection of Authenticated Cell Cultures (ECACC General Cell Collection, Salisbury, UK).

### 2.2. Preparation of Liposomes

The liposomes were prepared using the thin-film hydration method. Briefly, cholesterol, DPPC and DSPE-PEG (2000)-NH_2_, at molar ratios of 30:65:5, respectively, were dissolved in 4 mL of chloroform in a round-bottom flask. The chloroform was then evaporated using a rotary evaporator under vacuum at 50 °C for 15 min until a thin film was observed on the walls. Next, the lipid film was hydrated using 2 mL of the QD solution (at a concentration of 1 mg/mL) for 50 min at 60 °C. The liposomal solution was then sonicated for 2 min using a 40 kHz sonicator bath (Elma D-78224, Melrose Park, Illinois, USA) to obtain unilamellar vesicles and then extruded 30 times using 200 nm polycarbonate filters (Avanti Polar Lipids, Inc., Alabaster, Alabama, USA) to reduce the size of the formulations. The purification of the formulation was performed using centrifugation (Heraeus Megafuge 8 Centrifuge, Thermo Fisher Scientific Inc., Massachusetts, USA) at a speed of 17,850 rpm for 1 h at 4 °C. Finally, the collected fractions were resuspended in 1 mL PBS buffer and stored at 4 °C until use.

### 2.3. Measuring the Encapsulation Efficiency

Fluorescence spectra of the prepared QD-liposomes were determined using a fluorescence spectrophotometer at room temperature. The fluorescent intensity was measured at an excitation wavelength of 400 and an emission wavelength of 530 using a Synergy HTX microplate reader (Biotek, Winooski, Vermont, USA) following lysing the purified QD-liposomes with a surfactant (Triton X-100). A standard curve prepared using a pure solution of the graphene quantum dots was used to calculate the concentration of the encapsulated QDs.

### 2.4. Particle Size and Polydispersity Evaluation

The particle size and polydispersity index of liposomes encapsulating QDs were measured at 25 °C using DynaPro NanoStar (Wyatt Technology Corp., Santa Barbara, CA, USA) and Zetasizer Nano Zs instrument (Malvern Instruments Ltd., Malvern, UK). The tested sample was prepared by diluting 15 µL of liposomes in 1 mL of PBS.

### 2.5. Estimating Phospholipids Content of the Prepared QD-Liposomes

The total phospholipid content of the prepared QD-liposomes was measured colorimetrically using the Stewart assay [26]. A 50 µL volume of the prepared liposomes was placed in a round-bottom flask and evaporated using a rotary evaporator at 50 °C, under vacuum for 10 min. A 1 mL volume of chloroform was added, and the flask was sonicated for 20 s to release the phospholipids from the liposomal structure. A 200 µL volume of this mixture was added to a Pyrex tube, along with 1.8 mL more chloroform and 2 m of ammonium ferrothiocyanate L. Next, the tube was sonicated for 20 s and centrifuged at 1000 rpm for 10 min. The bottom layer was collected, and the absorbance values were recorded using UV–vis spectroscopy (Amax = 485 nm). Three replicates for each sample were measured.

### 2.6. Zeta Potentials

The zeta potential of the QDs and synthesized liposomes was measured using Malvern Zetasizer Nanomachine (Worcestershire, UK). The samples were prepared by diluting 15 µL of liposomes and QDs in 1 mL of distilled water.

### 2.7. Determination of Cell Viability

Human colorectal carcinoma cells (HCT116) were seeded in a 6-well plate at a concentration of 6 × 10^5^ cells/well and incubated overnight. This was followed by replacing the media, and the plates were exposed to ultrasound sonication using LFUS 60 s in a 35 kHz sonication bath. Nonsonicated plates were used as a control. The plates were incubated for a further 2 h. The cells were then detached using Trypsin EDTA and the percentage of cell viability was recorded using the Trypan Blue dye exclusion method.

### 2.8. Cellular Internalization Studies

For cellular internalization studies, the human colorectal carcinoma cells (HCT116) were cultured in DMEM medium supplemented with 10% FBS and 2 mM L glutamine using sterile coverslips inside 6-well plates. Following 24 h of incubation at 37 °C (5% CO_2_) in a humidified atmosphere, the cells were treated with 50/µL mL of cell culture media of either free QDs or QD-liposomes. Ultrasound-treated plates were subsequently irradiated with a 35 kHz ultrasonic bath for 60 s. The plates were then incubated for an additional 1 h. Next, the cells were washed with PBS and fixed with 4% paraformaldehyde, washed three times again with cold PBS and stained with diamidino-2-phenylindole blue dye (DAPI) to visualize the nucleus of the cells. The fluorescent images of QDs were captured using a confocal microscope with a 520/50 nm filter (Nikon eclipse Ti Melville, New York 11747-3064, USA.).

### 2.9. Transmission Electron Microscopy (TEM)

Samples were prepared by applying a 3 μL drop of the liposomes to a cleaned plasma thin holey carbon 400-mesh copper grid. After 30 min, the excess solution was removed using angled filter paper blotting. A drop (30 μL) of deionized water on a Parafilm was used to wash the grid by gently touching the surface of the grid, which was followed by filter paper blotting. The washing and blotting steps were performed two times, each with a fresh drop of deionized water. A drop (20 μL/drop) of 1% (*w*/*v*) uranyl acetate substitute solution was applied on a Parafilm, and the grid was placed facing down on the drop for 30 s. The sample was air-dried at room temperature following the removal of the excess stain. High-resolution transmission electron microscopy (HRTEM) and scanning transmission electron microscopy (STEM) images were obtained using a Talos F200X scanning/transmission electron microscope with a lattice-fringe resolution of 0.14 nm at an accelerating voltage of 200 kV equipped with a CETA 16M camera. All the relevant areas were marked using bright field imaging mode at spot size 5 and later scanned using the STEM-HDAAF mode at spot size 9 with a screen current of 60 pA. The analysis was performed at a low electron dose by collecting the high-angle dark-field signal using an annular detector. The data were analyzed using the Velox analytical software.

### 2.10. Statistical Analysis

The differences between the results were compared using a two-tailed *t*-test with the assumption of unequal variances. Two values were considered significantly different when *p* ≤ 0.05.

## 3. Results and Discussion

Characterization of the Prepared QD-Loaded Liposomes

**Figure 1:**
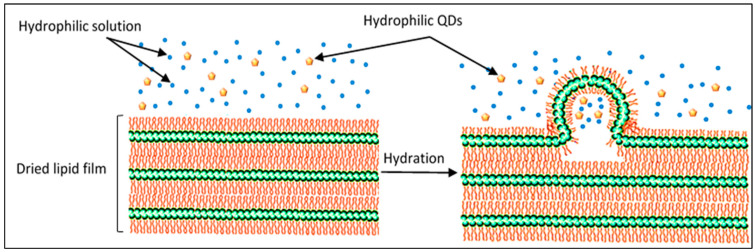
Scheme of the hydration of the dried lipid film and liposome formation. During this process, hydrophilic solutions and QDs are encapsulated inside the core of the formed liposomes.

The size of the different nanoparticles was determined using dynamic light scattering (DLS). The average size of the green graphene QD particles used in this study was 6 nm. The small size of the QDs allows their encapsulation inside the liposomes. As seen in Figure 1, the QDs used in this study are hydrophilic particles and, therefore, are expected to be encapsulated in the core of these nanocarriers, during the hydration process of liposomal formation, allowing extra space for encapsulation compared to the space provided inside the hydrophobic lipid bilayer.

**Figure 2:**
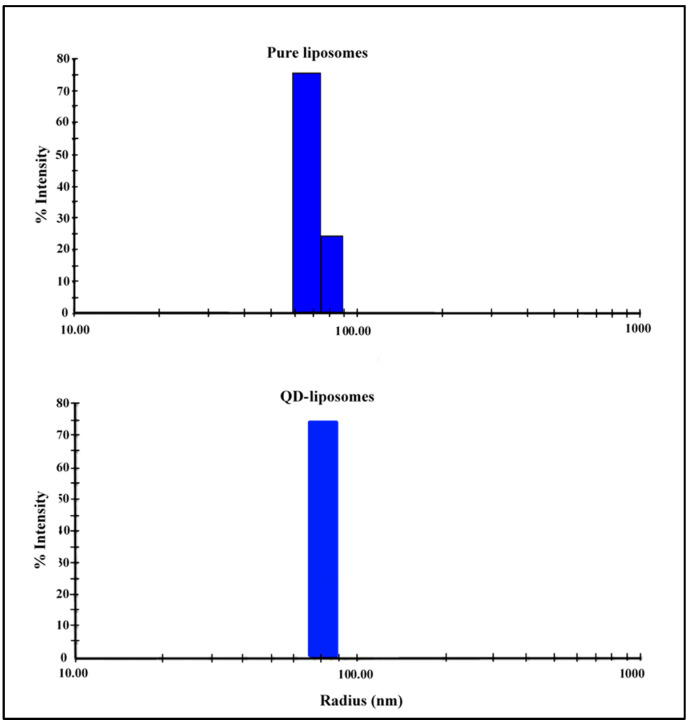
Size distributions of pure liposomes and QD-liposomes.

Three replicates were used for each sample, and the results showed that there was no significant difference in size between the pure liposomes and the QD-liposomes, with an average radius of 85 ± 9.71 nm and 86 ± 1.35 nm, respectively (*p* = 0.851). Both types of liposomes showed acceptable polydispersity percentages (Pd%) of 9.8% and 8.6% for the pure liposomes and QD-liposomes, respectively (Figure 2). Liposome size is the most crucial characteristic of liposomes. This is because, for an injectable form, liposome size should not exceed 200 nm in diameter to benefit from the EPR effect caused by the leaky vasculature surrounding tumors. The size of the liposomes also affects their blood circulation time and biodistribution, with larger liposomes (liposomes in the 400–500 nm size range) having higher clearance rates from the blood compared to 90–100 nm liposomes [27]. Therefore, the prepared QD-liposomes are within the recommended size as suitable nanocarriers delivering QDs to the cancer cells. While previous studies have reported no significant change in the hydrodynamic size of the liposomes following encapsulation [19,28,29,30], other studies have reported an increase in the size of these nanovehicles when they encapsulate QDs [31,32]. The differences in the reported findings could be due to different liposomal formulations and preparation techniques used among studies. Furthermore, encapsulating hydrophobic molecules, which are retained within the outer lipid bilayer, may increase the hydrodynamic size of the liposomes.

Measuring the size of the liposomes is a sensible indicator of the stability of liposomal suspensions [33,34]. To test the stability of these carriers, the size of the prepared QD-liposomes was recorded following their incubation at 37 °C in fetal bovine serum medium for 24 h. Previous studies have shown that the stability of nanocarriers in serum could predict their in vivo blood circulation [35,36]. Our results showed no significant difference in the size of the liposomes, showing an average radius of 86 ± 1.35 nm before incubation and 85 ± 0.32 nm following 24 h of incubation (*p* = 0.854). This confirms that the prepared liposomes are stable and can retain the integrity of their structure when circulating in the bloodstream. Chu et al. [37] showed that hydrophilic QDs remain trapped inside the liposomes following their storage at 4 °C for 609 days. This suggests that QD encapsulation inside the liposomes not only prevents their release from the liposomes but also reinforces the structure of the liposomes. In addition, crafting the liposomes with long chains of a hydrophilic polymer such as polyethylene glycol (PEG), also known as stealth liposomes, provides steric stabilization, reduces liposomal aggregation and gives rise to strong intermembrane repulsive forces. This improves the stability and pharmacokinetics of the QD-liposomes [38].

**Figure 3:**
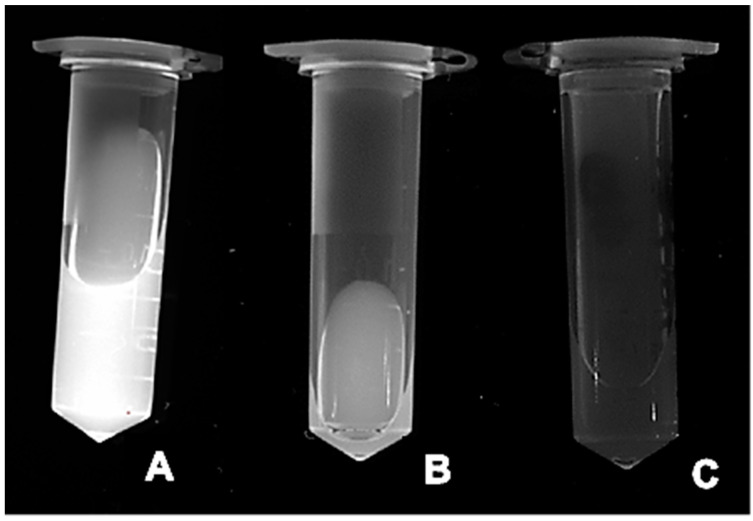
Images of (**A**) QDs, (**B**) QD-liposomes following centrifugation and (**C**) empty liposomes following centrifugation. All images were captured by placing the samples under a UV light.

To examine whether encapsulated QD particles inside the core of the liposomes preserved their fluorescence properties, the free QD solution, as well as QD-liposomes and empty liposomes, previously purified using centrifugation, was placed under a UV lamp (Figure 3). The empty liposomes were used as a negative control with no fluorescence signals. The strongest fluorescence signal was emitted from the QD solution. QD-liposomes also emitted high fluorescence but slightly lower than that of the QD solution. The phospholipids content of the prepared QD-liposomes, measured using a DPPC calibration curve, showed a value of 5.91 ± 0.17 mg/mL. QD molecules are present inside this phospholipid bilayer, which may act as a membrane barrier. This confirms that the QDs were successfully encapsulated inside the core of the liposomes and preserved their fluorescence properties. Furthermore, the prepared QDs showed an encapsulation efficiency of 30.9%. Previous studies have also shown that the encapsulation efficiency of QD-liposomes, prepared using the thin-film hydration method, is less than 40% [17,39].

**Figure 4:**
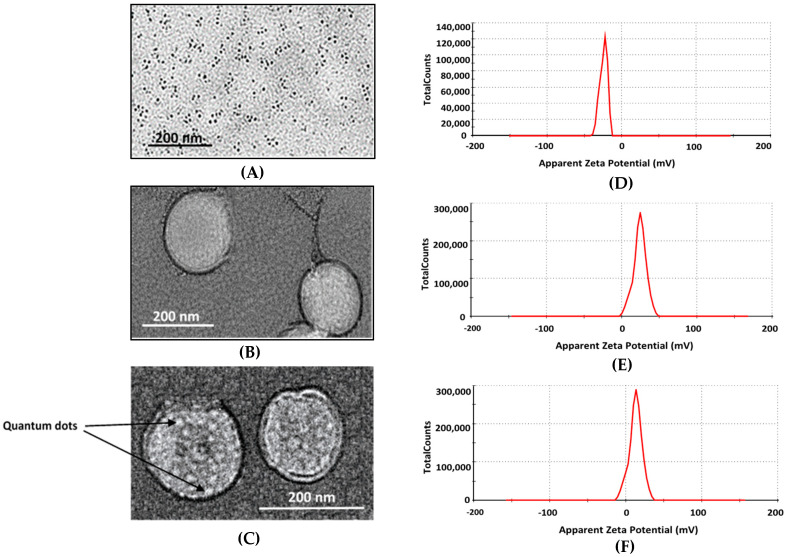
TEM images of free green graphene QDs (**A**), free liposomes (**B**) and QD-liposomes (**C**), as well as zeta potentials distribution for the free QDs (**D**), free liposomes (**E**) and QD-liposomes (**F**).

To further confirm the structure of the prepared liposomes and their encapsulation of QD nanoparticles inside their core, TEM images of both the pure and QD-liposomes, as well as the free QDs, were obtained (Figure 4). The images showed that QDs located inside the core of the liposome were successfully detected, indicating their efficient loading. Generally, intact spherical liposomes were formed, and no physical deformation of the liposomes was observed following QD encapsulation. This is in agreement with previous studies that have also shown a successful encapsulation of both hydrophilic and hydrophobic QDs inside liposomes with no effect on the shape of these nanocarriers [16,19,29,40,41,42].

Zeta potential is a measurement of the effective charge on the surface of nanoparticles. The zeta potential measurements showed that the surface of the free green graphene QDs was negatively charged, showing an average of −20.23 ± 2.53 mV. A significant shift in zeta potential value was recorded when encapsulating the QDs inside the stealth liposomes from −20.23 ± 2.53 mV to 6.98 ± 0.323 mV, giving evidence that QDs particles have been successfully loaded inside the liposomes. The surface charge of the pure liposomes, with no QD encapsulation, was 12.14 ± 0.143 mV (Figure 4). The loading of QDs reduced the zeta potential value of the stealth liposomes from 12.14 ± 0.143 mV to a lower positive value of 6.98 ± 0.323 mV. Other studies have shown that QD encapsulation inside the liposomes reduces the zeta potential of the liposomes [18,39]. The reported reduction in zeta potentials could be due to the presence of nonionic materials in the liposomal bilayer, which may shield the surface potentials. Other studies encapsulating hydrophobic QDs with the lipid bilayer of the liposomes showed an increase in the negative charge following QD encapsulation. The change in the zeta potentials did not affect the stability of the QD-liposomes following their incubation at 37 °C for 24 h showing no significant change in the size, as mentioned earlier. In addition, the positive surface charge of the QD-liposomes increases cellular liposome uptake, as previously reported by Bothun et al. [40] and Al-Jamal et al. [43].

Xiao et al. [44] showed that both normal and cancerous cells have the ability to passively internalize large amounts of QDs. Therefore, delivering QDs specifically to tumors can be achieved by blocking or minimizing the passive delivery of QDs. In order to evaluate the benefits of QD-liposomes in controlling the cellular uptake of QDs, HCT116 cells were incubated with either free QDs or QD-liposomes. Furthermore, we applied LFUS on the cells to investigate the effect of ultrasound on the cellular uptake of free and encapsulated QDs. Sonication of the human colorectal carcinoma cells (HCT116) with low-frequency ultrasound (35 kHz) had no significant effect on cell viability, showing no difference in the percentage of the viable cells when compared with the nonsonicated control cells (Table 1). This indicated that the application of LFUS on the cells did not result in destroying the cancer cells (*p* > 0.05). Previous in vitro studies have also shown that ultrasound irradiation did not result in cellular destruction [45,46]. Thus, LFUS triggering of content release from the liposomes is an effective and safe modality.

**Figure 5:**
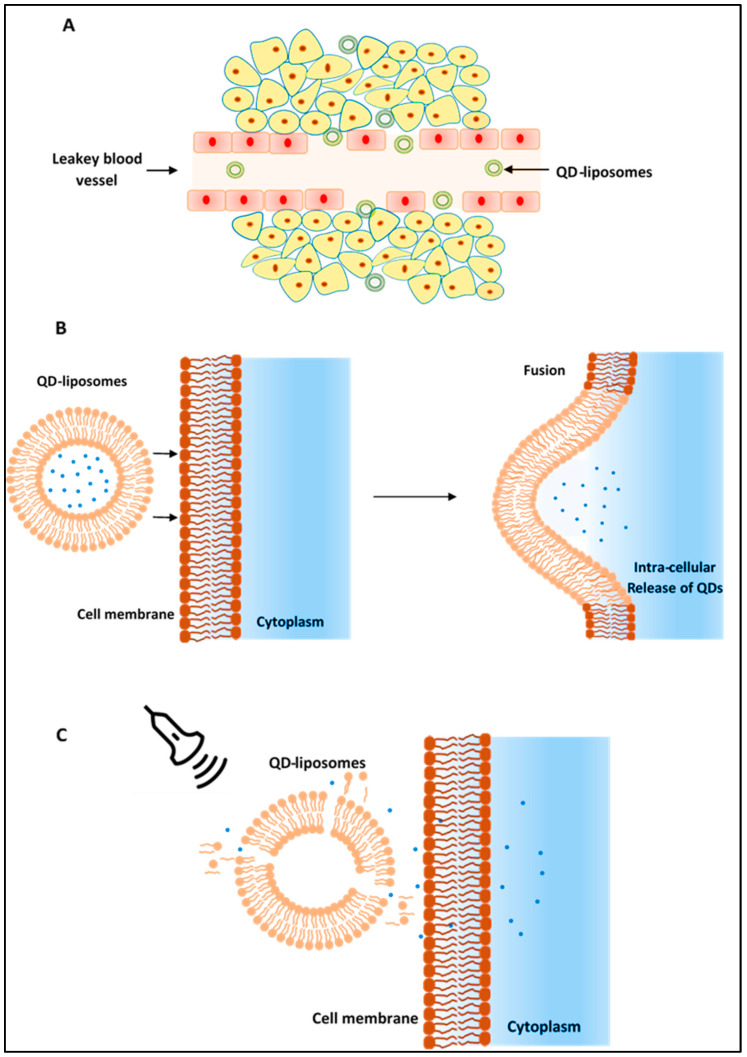
(**A**) QD-liposomes are able to extravasate through the leaky vessels surrounding the cancer cells (EPR effect) allowing their accumulation at the tumor site. (**B**) QD-liposomes are then taken up by the cells through their fusion with cellular membranes. (**C**) Ultrasound can be applied on the tumor site to trigger the release of QDs encapsulated inside the liposome and enhance their uptake by the cancer cells mainly through the sonoporation effect produced by the ultrasound.

Injecting free QDs into the bloodstream means that uncontrollable amounts of QDs are delivered into the cells, which significantly increase their toxicity and may produce undesirable fluorescent background noise. Therefore, by encapsulating QDs inside the liposomes, better control of the delivered QDs is expected. The larger size of the liposomes compared to QDs means that they are not able to pass through the intact blood vessels of the healthy tissues but are able to extravasate through the leaky surrounding the tumor cells and accumulate inside these diseased tissues only. This is known as passive targeting of tumors. These liposomes are then able to bind and fuse with cellular membranes of the cancer cells, allowing the uptake of their loads of QDs. However, cellular uptake of liposomes through their fusion with cellular membranes is a low process. Therefore, we are proposing the use of LFUS as a triggering mechanism to trigger and control QD release from the liposomes and enhance their uptake by the cells due to the sonoporation effect produced by the ultrasound (Figure 5).

**Figure 6:**
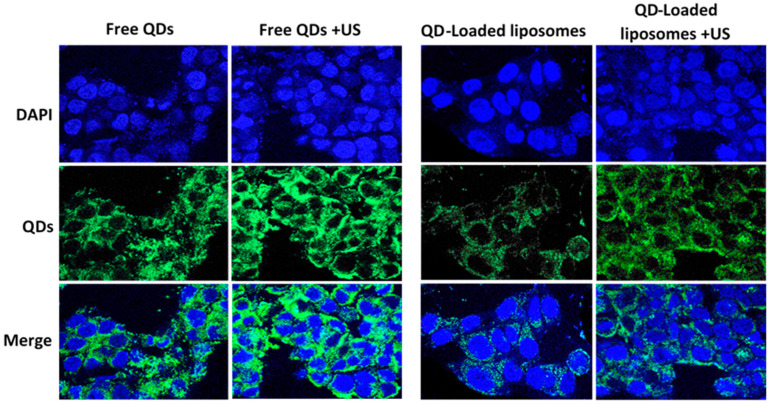
HCT116 cells incubated with free QDs or QD-liposomes with and without sonication with ultrasound (US) for 1 min at 37 °C. The first row only shows the nuclei of the cells stained with DAPI (blue). An argon laser (520/50 nm) was used for QD excitation producing a green fluorescence (second row). The third row shows merged images of both the nuclei and QDs present inside the cells.

As seen in Figure 6, the fluorescent microscopic images showed that QD encapsulation inside the liposomes significantly reduced the amount of QDs present inside the cells. This is in agreement with Bruun and Hille [19], who showed that the diffusion rate of free QDs is much faster than that of QD-liposomes due to the difference in size. The small size of the QDs allows them to be taken up by the cells in large quantities through nonspecific adsorption and nonspecific binding to different intracellular membrane compartments leading to ligand- and receptor-independent cell uptake and nonspecific internalization to the cytosol [47]. This results in the uptake of large quantities of free QDs in a short period of time by both healthy and cancerous cells due to their low specificity. QD-liposomes, on the other hand, interact with the cells by adhering to the cell surface and subsequently binding to the cells [48]. Following such binding, the liposomes are absorbed or internalized into the cells releasing their content inside the cytosol. Our QD-liposomes were positively charged, which enhances their association with the negatively charged cellular membranes through electrostatic interactions, which may increase their uptake by the cells compared to negatively charged QD-liposomes.

As shown in Figure 6, sonication with LFUS resulted in stronger fluorescence signals, indicating a significant increase in the amount of QDs present inside the cells. This is due to the enhancement of QD transport by passive diffusion through the cellular membrane. Ultrasound imposes mechanical stress on cellular membranes due to cavitation events, leading to the formation of transient wounds or pores on the membrane [49]. This allows more QD particles to pass into the cell. This is in agreement with a previous study by Thein et al. [50], who reported that ultrasound irradiation enhanced cellular uptake of QDs through sonoporation, showing that pore size and the subsequent cellular uptake of QDs increased linearly with the increase in ultrasound pressure. Turcanu and coworkers [51] have also shown that sonication with ultrasound enhanced the passage of QD across the intestinal mucosa. The latter acts as a selective barrier to the permeation of materials. This was later confirmed in a more recent study by Stewart et al. [52], who also showed that applying focused ultrasound enhanced the delivery of QDs to the small intestine during in vivo experiments using porcine models. In addition to the sonoporation effect, ultrasound waves also create changes in intracellular calcium concentration (Ca^2+^). This results in affecting the tight junction of the endothelial cells and disturbing cell barrier permeability [53]. As seen in Figure 6, cells incubated with QD-liposomes also showed a higher level of fluorescence compared to the nonsonicated cells. This is because liposomal membranes have a similar structure to cellular membranes. Thus, sonication with LFUS and the cavitation-mediated pore formation (sonoporation) on both the phospholipid walls of the cells and the liposomes result in both enhanced liposome uptake by the cells and QD release from the encapsulating liposomes. We showed in a previous study that liposomes crafted with PEG or pegylated liposomes are more sensitive to LFUS compared to nonpegylated liposomes [54]. This is because the incorporation of PEG with the lipid bilayer of the liposomes enhances the ability of the LFUS to permeabilize these liposomes, since it is more likely for the PEG polymer to be ejected out of the phospholipids bilayer to form smaller micelles upon exposure to ultrasound waves. This results in triggering the release of the loaded QDs, reflected in the increase in fluorescence intensity inside the sonicated cells. These findings suggest that combining LFUS with QD-liposomes ensures an effective delivery and release of the QDs to the tumor site while reducing the uptake of QDs by the healthy tissues.

## 4. Conclusions

In this work, we successfully encapsulated hydrophilic QDs inside stealth liposomes. TEM images showed that QDs were mainly retained within the aqueous core of the liposomes. We also showed that low-frequency ultrasound is an effective method to release QDs encapsulated inside the liposomes in a spatially and temporally controlled manner. Overall, LFUS-triggered QD-liposomes show promise in tumor imaging applications with promising potentials as a theranostic system for diagnostic and therapeutic functions.

## Figures and Tables

**Table 1:** Cell viability following 60 s of sonication using LFUS (35 kHz).

	Control	Sonicated	*p*-Value
Viability %	96.9%	97.74%	0.862
Std. Dev	2.45	1.28	

## Data Availability

The data used to support the findings of this study are included within the article.
